# Localization and Classification of Paddy Field Pests using a Saliency Map and Deep Convolutional Neural Network

**DOI:** 10.1038/srep20410

**Published:** 2016-02-11

**Authors:** Ziyi Liu, Junfeng Gao, Guoguo Yang, Huan Zhang, Yong He

**Affiliations:** 1 College of Biosystems Engineering and Food Science, Zhejiang University, 866 Yuhangtang Road, 5 Hangzhou 310058, China; 2 Department of Plant Protection, Zhejiang University, 866 Yuhangtang Road, 5 Hangzhou 310058, China

## Abstract

We present a pipeline for the visual localization and classification of agricultural pest insects by computing a saliency map and applying deep convolutional neural network (DCNN) learning. First, we used a global contrast region-based approach to compute a saliency map for localizing pest insect objects. Bounding squares containing targets were then extracted, resized to a fixed size, and used to construct a large standard database called *Pest ID*. This database was then utilized for self-learning of local image features which were, in turn, used for classification by DCNN. DCNN learning optimized the critical parameters, including size, number and convolutional stride of local receptive fields, dropout ratio and the final loss function. To demonstrate the practical utility of using DCNN, we explored different architectures by shrinking depth and width, and found effective sizes that can act as alternatives for practical applications. On the test set of paddy field images, our architectures achieved a mean Accuracy Precision (mAP) of 0.951, a significant improvement over previous methods.

Pest insects are known to be a major cause of damage to the world’s commercially important agricultural crops^1^. Since the 1960s, integrated pest management (IPM) has become the dominant pest control paradigm, being endorsed globally by scientists, policymakers, and international development agencies^2^. IPM requires the monitoring of pressures from different pest insect species, allowing the development of optimal pesticide recommendations that promote favorable economic, ecological and sociological consequences^3^. Therefore, the accurate recognition and quantitation of pests is of central importance for the effective use of IPM^4^. However, most current monitoring practices are expensive and time-consuming, as they require IPM professionals to manually collect and classify specimens in the field, impeding the extension of this technology to regions who lack this technical support, including most of the developing world^2^,^5^. More inexpensive methods are therefore required, and automated systems based on computer vision and machine learning has emerged as an exciting technology that can be applied to this issue^6^.

The objective of an automated visual system is to provide expert-level pest insect recognition with minimal operator training^7^. There are several fundamental challenges emerged in the pursuit of this objective. These include: (1) wide variations in the positioning of pest insect objects and being able to distinguish the insect objects from varying degrees of background clutter, (2) the significant intra-class difference and large inter-species similarity that exists for many species, (3) a requirement for a fast collection and interpretation of data to allow rapid responses, particularly when large numbers of pests are detected. In past decades, such challenges motivated many research groups to develop practical imaging systems for this purpose. In the remainder of this section, we first give a brief review of the current state of the field, and then present our justification for our work on this problem. Most of the previous research can be described by a framework composed of two modules^8^: (1) *representation* of the pest insect images: the computer vision-based feature extraction, and their preprocess (*i.e.*, obtaining and organizing effective information from the features). (2) the subsequent architecture of *machine learning*: the computational learning models implemented to classify the represented features.

Early research on pest insect recognition used global low-level image representation based on color, texture or geometric invariants, such as color histogram and Gray-Level Co-occurrence Matrices (GLCM)^9^, geometric shape (eccentricity, perimeter, area, etc.)^10^,^11^, Hu moment invariants^12^, eigen-images^13^, wavelet coding^14^ or other relatively simple features^15^,^16^,^17^. The rationale for this approach is that the pest recognition problem can be formulated as a problem requiring the ability to match appearance or shape. The development of programs including the automated bee identification system (ABIS)^16^, digital automated identification system (DAISY)^13^ and species identification, automated and web accessible (SPIDA)-web^14^ demonstrated the early proof-of-concept of the applicability of this approach, and a slew of research followed. It was shown that these applications could be highly effective under ideal imaging conditions (*e.g.*, no occlusion, controlled lighting, and a single pose of top view etc.), resulting in good performance for relatively small database sizes with small inter-object similarity. However, their selected features were not detailed, and only provided the principal contours and textures of the images, insufficient to allow the learning models to handle pest species with much finer distinctions. Moreover, most of these systems require direct manual manipulation (*e.g*., manually identifying the key image features), which is as expensive as the traditional recognition procedure. For systems that need to recognize thousands of samples in the field, the requirement for manual operation on images makes this process slow, expensive, and inefficient.

To address such problems, researchers began using local-feature based representation of pest insect images to allow learning with much less user interaction^18^,^19^,^20^,^21^,^22^,^23^,^24^. The most popular of these local feature-based methods are based on the bag-of-words framework^25^ and work by partitioning pest images into patches with local operators (LBP^26^, SIFT^27^, HOG^28^, etc.), encoding each using a dictionary of visual words, and then aggregating them to form a histogram representation with the minimum encoding length. This parts-based representation is beneficial for recognizing highly articulated pest insect species having many sub-parts (legs, antennae, tails, wing pads, etc.). Meanwhile the minimum encoding length can build a compact representation more robust to imagine difficulties due to background clutter, partial occlusion, and viewpoint changes^29^. However, they rely on the careful choice of features (or good patch descriptors), and a sophisticated design for the preprocess procedure (*i.e.* ways to aggregate them). If incomplete or erroneous features are extracted from paddy field images, in which quite a number of pixels might be in background clutter, the subsequent classifier would be dominated by irrelevant variations of background^20^. If an off-the-shelf preprocess of the extracted features is incapable of refining meaningful fine distinctions, the individuals of highly similar species would not be able to be distinguished by the learning models^30^. Furthermore, wide variation in intra-species and pose usually requires a sufficient number of training samples to cover their whole appearance range^8^, a challenge that most applications fail to meet.

Ad-hoc feature extraction and preprocessing can, to a considerable extent, help to mitigate the above problems, for example, by using a novel task-specified feature^31^ or an adaptive coding strategy^32^. Such improvements exhibited satisfying performance for rather fine-grained identification tasks. For example, the recent report claimed excellent results for a complicated arthropod species with differences so subtle that even human experts have difficulty with classification^31^,^33^. These efforts are important, but still rely on prior expert knowledge and human labor; if task-specified designs must be devised for each new category of pest insects, achieving generalization performance will require an overwhelming amount of human effort^34^.

The previous work therefore lead us to the following questions: what are the ideal visual features for a pest insect recognition task and what is the optimal way to organize discriminative information from these features to easily apply a learning model, with minimal human intervention.

Recently, deep convolutional neural networks (DCNNs) have provided theoretical answers to these questions^34^,^35^, and have been reported to achieve state-of-the-art performance on many other image recognition tasks^36^,^37^,^38^. Their deep architectures, combined with good weight quantization schemes, optimization algorithms, and initialization strategies, allow excellent selectivity for complex, high level features that are robust to irrelevant input transformations, leading to useful representations that allow classification^39^. More importantly, these systems are trained *end to end*, from raw pixels to ultimate categories, thereby alleviating the requirement to manually design a suitable feature extractor.

Inspired by the success of DCNN, we attempted to test variations in DCNN for its ability to overcome common difficulties in a pest recognition task. For our test, we used the classification of 12 typical and important paddy field pest insect species. We selected a network structure similar to a well-known architecture AlexNet^36^, and utilized its GPU implementation. We addressed several common limits of these systems, as follows:

1. the requirement of a large training set; we collected a large amount of natural images from Internet.
2. input images of fixed size; we introduced a recently developed method, “global contrast based salient region detection”^40^, to automatically localize and resize regions containing pest insect objects to an equal scale, and constructed a standard database *Pest ID* for training DCNN.
3. optimization difficulties; we varied several critical parameters and powerful regularization strategies, including size, number and convolutional stride of local receptive fields, dropout ratio^41^ and the final loss function, to seek the best configuration of DCNN.

In performing these tests, we were able to address DCNN’s practical utility for pest control of a paddy field, and we discussed the effects of reducing our architecture on runtime and performance. This method achieved a high mean Accuracy Precision (mAP) of 0.951 on the test set of paddy field images, showing considerable potential for pest control.

## *Pest ID* Database

### Data Acquisition

Our original images were collected from image search databases of Google, Naver and FreshEye, including 12 typical species of paddy field pest insects with a total of over 5,000 images. To avoid duplicates and cover a variety of poses and angles, images of each species were manually refined by three censors. Pixel coordinates of all selected images were normalized to [0, 1].

### Construction of *Pest ID*

We adopted a net architecture similar to AlexNet^36^ (see section **Overall Architecture**), which is limited to input images of 256 × 256 pixels. This required changing the size from the original images by careful cropping that maintained a centered pest insect object. Thus, a localization method was required.

#### Salient region based detection

**Figure 1:**
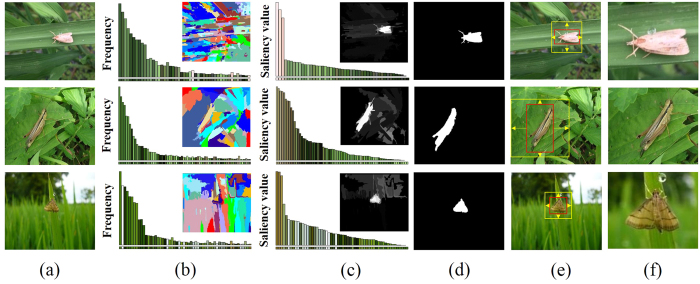
Examples of constructing *Pest ID*. (**a**) original images, in which the first and the third rows are provided by Guoguo Yang, and the second row courtesy of Keiko Kitada. (**b**) segmented regions and the corresponding color histograms. (**c**) saliency maps and saliency value of each region. (**d**) GrabCut^45^ segmentation results initialized from the thresholded saliency maps. (**e**) localization results, in which tight bounding boxes (red) containing pest insect objects are extended to squares (yellow). (**f**) *Pest ID* images.

In the original set of images we observed that pest insect objects usually occupy highly contrast color regions relative to their backgrounds (Fig. 1(a)). Many physiological experiments and computer vision models have proved such regions have a higher so-called saliency value than that of their surroundings, which is an important step for the object detection^42^,^43^. Thus we applied a recently developed approach “global contrast based salient region detection”^40^ to automatically localize the regions of pest insects in given images, as detailed below.

Shown in Fig. 1, the original images (Fig. 1(a)) are first segmented into regions using a graph-based image segmentation method^44^, and then color histograms are built for each region (Fig. 1(b)). Due to the efficiency requirement, each color channel (RGB) of the given images is quantized to have 10 different values, which reduces the number of all colors to 10^3^. For each region *r*_*k*_, the saliency value *S*(*r*_*k*_) is computed to represent its contrast to others,


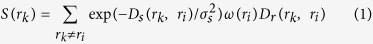


Where,


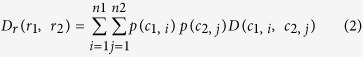


where *ω*(*r*_*i*_) is the number of pixels in *r*_*i*_, *D*_*s*_ and *D*_*r*_ are respectively the spatial distance and color space distance metric between two regions, and *σ*_*s*_ controls the strength of the spatial weighting. For each region *r*_*k*_, its salience value benefits from its spatial distance to all other regions, and here a large value of *σ*_*s*_ (0.45) is used to reduce the effect of this spatial weighting so that contrast to father regions would contribute more to the saliency value of current region. Note that in *D*_*r*_, based on the color histogram (Fig. 1(b)), the probability *p*(*c*_*m,i*_) of each color *c*_*m,i*_ among all *n*_*m*_ colors in the *m*-th region *r*_*m*_ is considered for the original color distance *D, m* = 1, 2, giving more weight to the dominate color difference. These steps are used to obtain the maps (Fig. 1(c)) indicating the saliency value of each region. We can see from these saliency maps that the regions representing pest insect objects are of higher value compared to background.

#### GrabCut based localizatiohn

The computed saliency maps are then used to assist a segmentation of pest insect objects, a key step to the subsequent localization. A GrabCut^45^ algorithm is initialized using a segmentation obtained by thresholding the saliency maps using a fixed threshold *th* (0.3) which is chosen experimentally to give the localization accuracy of over 0.9 in a subset of the original images (see details in section **Localization Accuracy of Saliency Detection**). After initialization, an iteration of 3 times of GrabCut is performed, which gives the final results of the rough segmentation of pest insect objects (Fig. 1(d)). With these segmentation results, the bounding boxes containing the pest insect objects are extended as squares (Fig. 1(e)), and then cropped from their original images. Finally, all cropped regions are resized as 256 × 256 (Fig. 1(f)) for constructing the standard database *Pest ID*. Details of *Pest ID* are shown in Table 1, and its online application is being built.

## Deep Convolutional Neural Network

### Overall Architecture

**Figure 2:**
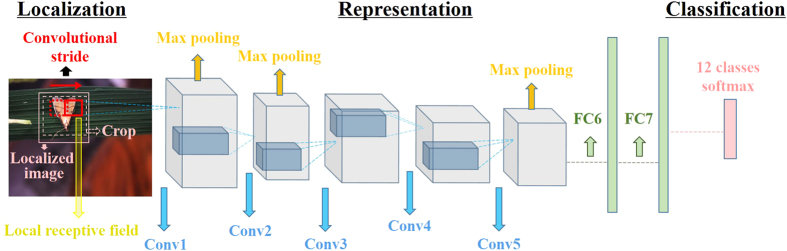
Overall architecture of the model. After saliency detection, a 227 × 227 crop of the localized image is presented as the input. It is convolved in the first convolutional layer (Conv1) with local receptive fields, using a convolutional stride of fixed step. The results are then represented in vector form through other 4 convolutional layers (Conv2–5) which are with 3 max pooling layers, and two fully connected layers (FC6, FC7). The final layer is a 12-way softmax function for classification. Original image courtesy of Junfeng Gao.

We implemented and altered the net architecture (Fig. 2) based on AlexNet^36^. This 8-layer CNN network can be thought of as a self-learning progression of local image features from low to mid to high level. The first five layers are called convolutional layers (Conv1-5), in which the higher layers synthesize more complex structural information across larger scales sequences of convolutional layers. Interleaved with the max pooling, they are capable of capturing deformable parts, and reducing the resolution of the convolutional output. The last two fully connected layers (FC6, FC7) can capture complex co-occurrence statistics, which drop semantics of spatial location. A final classification layer accepts the previous representation vector for the recognition of a given image. This architecture is appropriate for learning powerful local features from the complex natural image dataset^46^. A schematic of our model is presented below (see reference^36^ for more network architecture details).

### Training the Deep Convolutional Neural Network

Each input image is processed as 256 × 256 as previously. 5 random crops (and their horizontal mirrors) of size 227 × 227 pixels are presented to the model in mini-batches of 128 images. Each convolutional layer is followed by rectification non-linearities (ReLU) activation, and max pooling are located after the first (Conv1), second (Conv2) and fifth (Conv5) convolutional layers. The last layer (classification layer) has 12 output units corresponding to 12 categories, upon which a softmax loss function is placed to produce the loss for back-propagation. The initial weights in the net are drawn from a Gaussian distribution with zero mean with a standard deviation of 0.01. They are then updated by stochastic gradient descent, accompanied by momentum term of 0.8 and the L2-norm weight decay of 0.005. The learning rate is initially 0.01 and is successively decreased by a factor of 10 during 3 epochs, each of which consists of 20000 iterations. We trained the model on a single NVIDIA GTX 970 4GB GPU equipped on a desktop computer with a Intel Core i7 CPU and 16GB memory.

### Dropout

Overfitting is a serious problem in a network with a large set of parameters (about 60 million). The 12 classes in *Pest ID* used only 10 bits of constraint on the mapping from image to label, which could allow significant generation error^47^. Dropout^41^ is a powerful technique to address this issue when data is limited. This works by randomly removing net units at a fixed probability during training, and by using a whole architecture at test time. This counts as combining different “thinned” subnets for improving the performance of the overall architecture.

## Experiment and Analysis

### Localization Accuracy of Saliency Detection

Thresholded saliency maps present the initial regions for GrabCut^45^ segmentation and thus determine the final localization results. In order to comprehensively evaluate the effects of different threshold *th* on the localization accuracy, we varied this parameter from 0.1 to 0.9 in steps of 0.1. Note that in this evaluation, the correct localization result on each original image is defined by two restrictions: (1) the area difference between the localization box and the ground truth box less than 20% of the latter, (2) at least 80% localization region pixels belong to that of the ground truth region. The ground truth boxes of all original images were manually labelled before.

**Figure 3:**
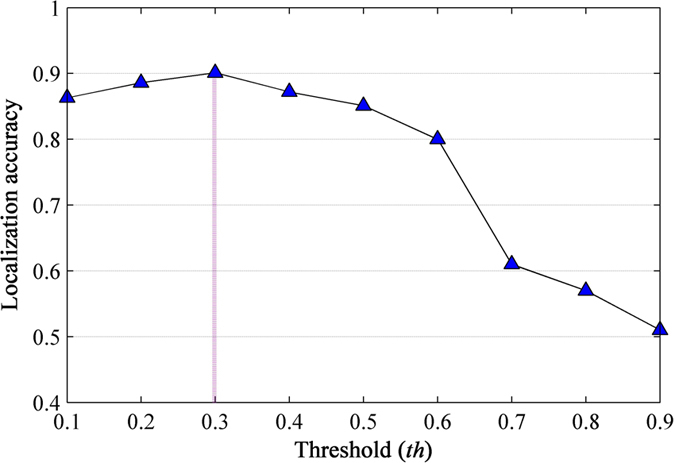
Localization accuracy under different threshold *th*.

**Figure 4:**
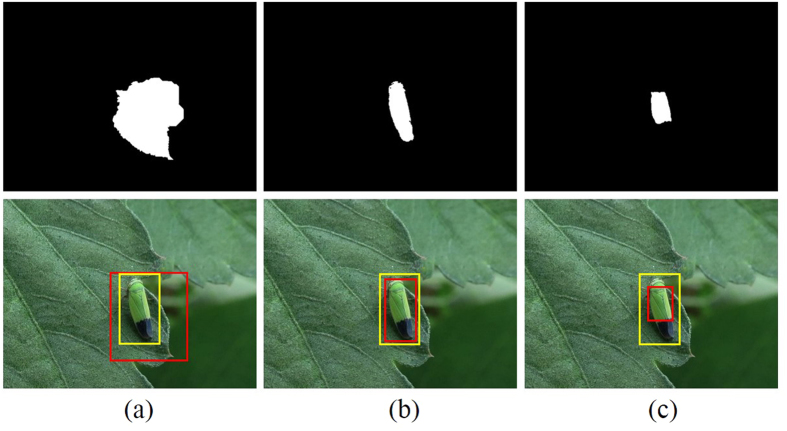
An example for visual comparison of localization results at different threshold *th*. (**a**) *th* = 0.1. (**b**) *th* = 0.3. (**c**) *th* = 0.5. Column 1: Thresholded saliency maps. Column 2: The ground truth boxes (yellow) and localization boxes (red) in the original images. Original image for this example courtesy of Masatoshi Ohsawa.

As shown in Fig. 3, the localization accuracy curve achieves its maximal value at the point of 0.3 where over 90% of localization results meet above restrictions. The visual comparison (see Fig. 4) illustrates that lower threshold values capture too much unwanted background (Fig. 4(a)), while one that is too high might be unable to highlight the whole target object (Fig. 4(c)). At the optimal point, there remains a fraction of pest objects that are not detected. We investigated these failure cases, and found that most of them could be attributed to the high background bokeh in the original images; when both the pest insect and their nearby regions are of high contrast to the distant regions, they have similar saliency. This can result in undesirable thresholded saliency maps including too many unwanted initial regions, making GrabCut difficult to segment pest insect objects.

Despite the weakness in the above special cases, this approach is still expected to be a promising tool for pest insect localization due to its low computation cost and simplicity^40^, which will be beneficial for practical applications. In the future, we plan to increase detection, using exhaustive search^39^ or selective search^48^, in the resulting saliency maps. This is necessary for generalizing the localization ability of saliency detection, and extension of the *Pest ID* database to contain more pest insect species.

### Optimization of the Overall Architecture

The overall architecture includes a number of sensitive parameters and optimization strategies that can be changed: (i) size, number, and convolutional stride of the local receptive fields, (ii) dropout ratio for the fully-connected layers, and (iii) the loss function in the final classification layer. In this section, we present our experimental results testing the impact of these factors on performance.

### The Role of the Local Receptive Fields

#### Size of local receptive fields

**Figure 5:**
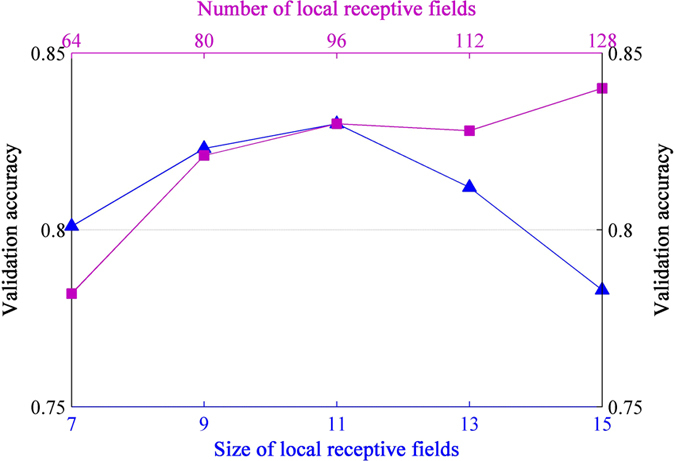
Effects of size and number of local receptive fields (first layer filters) on the validation accuracy. The testing of number of local receptive fields was based on their size being 11 × 11. About 25% of the images from each species in *Pest ID* were randomly selected for constructing the validation set, totaling 1210 images.

**Figure 6:**
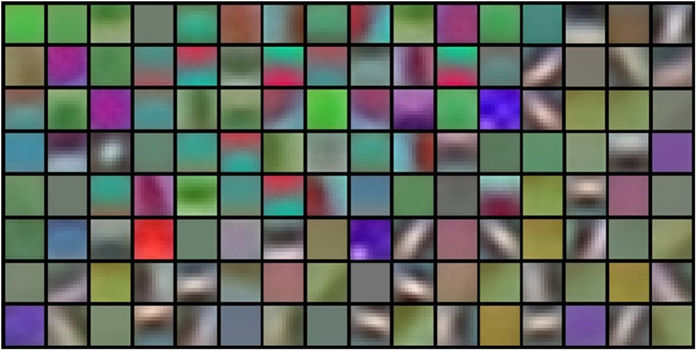
Visualization of local receptive fields. 128 local receptive fields of size 11 × 11 are projected to pixel space.

Local receptive fields are actually the filters in the first layer (see Fig. 2). Their size is usually considered to be the most sensitive parameter, upon which all the following works are built^49^. The ordinary choice of this parameter is in the range of 7 × 7 to 15 × 15 when the image size is around 200 × 200^50^. In this experiment, we ascertained that 11 × 11 works best for *Pest ID* images (see Fig. 5). The reason might be that the pest objects have similar scales and thus are rich in both structure and texture information. Normally, small receptive fields focus on capturing texture variation, while large ones tend to match structure differences. In this regard, our selected filters achieved the balance between these tendencies. For example, a round-shaped image patch can be recognized as an eye or spots using a suitable receptive field, but this recognition might be infeasible at a smaller or larger size. As illustrated in Fig. 6, these filters tend to produce biologically plausible feature detectors like subparts of pest insects.

#### Number of local receptive fields

A reasonable deduction could be that the net uses significantly fewer receptive fields than AlexNet^36^, because we have fewer classes compared to other tasks like Imagenet^51^. Unexpectedly, we still found that more local receptive fields led to better performance (Fig. 5). A possible explanation is that pest objects lack consistency in the same class due to the intra-class variability and the viewing angles (pose) difference. Thus more receptive fields are needed to ensure that enough variants for the same species can be captured.

#### Convolutional stride

**Figure 7:**
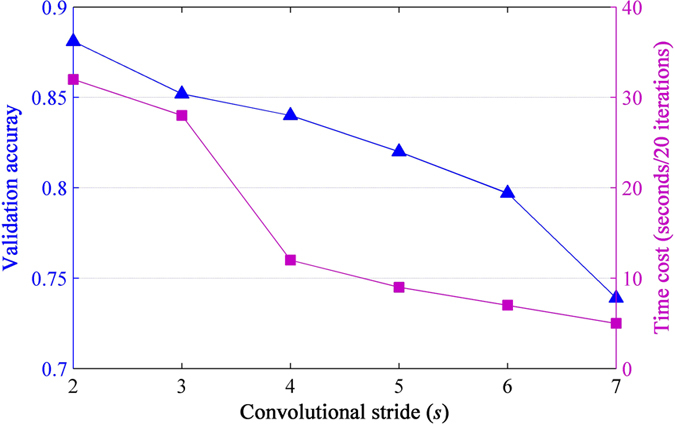
Effects of different convolutional stride of local receptive fields on validation accuracy and the corresponding time cost. The time is calculated for 20 iterations.

The convolutional stride *s* used in the net is the spacing between local image patches where feature values are extracted (see Fig. 2). This parameter is frequently discussed in convolutional operations^49^. DCNNs normally use a stride *s* > 1 because computing feature mapping is very expensive during training. We fixed the number of local receptive fields (128) and their size (11 × 11), and varied the stride over (2, 3, 4, 5, 6, 7) pixels, to investigate how much performance compromise costs in terms of time. Shown in Fig. 7, both validation accuracy and time cost show a clear downward trend with increasing step size as expected. For even a stride of *s* = 3, we suffered a loss of 3% accuracy, and saw bigger effects when using the larger ones. To achieve the trade-off with time cost, we adopted *s* = 3 that confers the smallest change in validation accuracy without significantly increasing the time of training.

### Effects of Dropout Ratio

**Figure 8:**
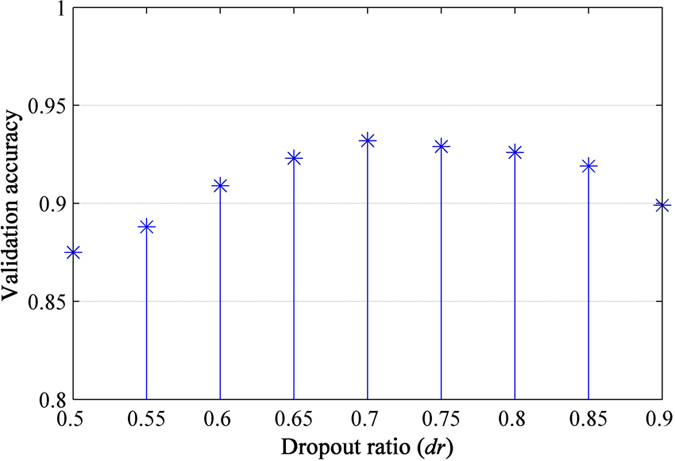
Effect of changing the dropout ratio on resulting validation accuracy.

Dropout has a tunable hyperparameter dropout ratio *dr* (the probability of deactivating a unit in the network)^41^. A large *dr* therefore means very few units will turn on during training. In this section, we explored the effect of varying this hyperparameter within the range between 0.5 and 0.9 which is most recommended^41^. In Fig. 8, we see that as *dr* increases, the error decreases. It becomes flat when 0.65 ≤ *dr *≤ 0.8 and then increases as *dr* approaches 1. Since dropout can be seen as an approximate model combination, a higher dropout ratio implies that more submodels are used. Thus, the network performs better at a large *dr* (such as 0.7). However, a too aggressive dropout ratio would lead to a network lacking sufficient neurons to model the relationship between the input and the correct output (such as *dr* = 0.9).

### Effects of the Loss function

**Figure 9:**
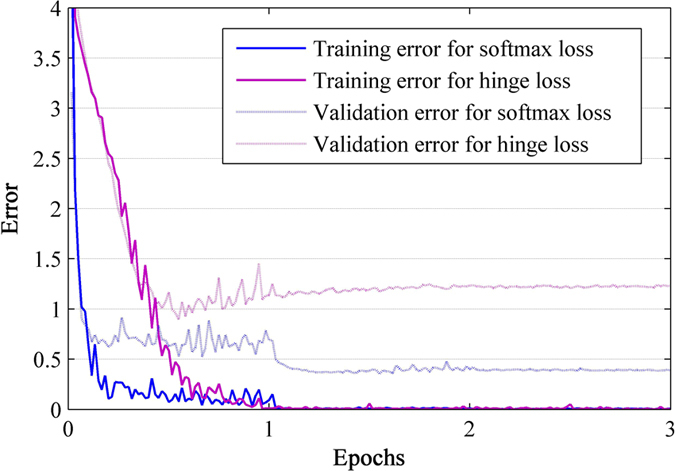
Training and validation errors for hinge loss (one-versus-all) or softmax loss as learning proceeds. The errors were computed over 3 epochs, of which each has 20000 iterations. Both learning processes used the same local receptive fields and drop ratio. The two loss functions were associated with a large L2-norm weight decay constant 0.005 (larger than that used in AlexNet^36^), which has proved to be useful for improving generalization of neural networks^55^. Under these settings, softmax and hinge respectively achieved 0.932 and 0.891 in validation accuracy.

The most popular loss functions used with DCNNs are logistic, softmax and hinge loss^52^. Here we investigated the effectiveness of softmax vs hinge (one-versus-all) for training (since logistic function is a derivative of softmax^53^, we did not test it here). Both functions were tested using the same learning setting (size, number and stride of local receptive fields of 11, 128 and 3, and a dropout ratio of 0.7), and a large L2-norm weight decay constant of 0.005 to prevent overfitting. Under these conditions, softmax slightly outperformed hinge loss (0.932 vs. 0.891 in validation accuracy). To explicitly illustrate the advantage of softmax, we plotted the learning procedures of these two functions in Fig. 9. It can be seen that learning with softmax allowed better generalization (similar training error but much smaller validation error than hinge), and converged faster.

Although on the *Pest ID* database softmax shows better results, this should now be adopted as the standard loss as our tested parameters are too limited. If *Pest ID* is augmented to include significantly more species, it will be necessary to re-address this issue.

### Practical Utility of the Model

From a practical standpoint, use of this strategy for paddy field applications requires that the model can execute in real-time and achieve rapid retraining by accepting new samples or additional classes. It is desirable, therefore, to seek approaches to speed up the models while still retaining a high level of performance. In this section, we focus on structural changes in the above overall architecture that enable faster running times with small effects on performance. In Table 2, we analyzed the performance and the corresponding runtime of our model by shrinking its depth (number of layers) and width (filters in each layer).

#### Ablation of entire layers

We first explored the robustness of the overall architecture by completely removing each layer. As shown in Table 2, removing the fully-connected layers (Type-2, 3) made only a slight difference to the overall architecture. This is surprising, because these layers contain almost 90% of the parameters. Removing the top convolutional layers (Tpye-4) also had little impact. However, removing the intermediate convolutional layers (Type-5, 6, 7) resulted in a dramatic decrease in both accuracy and runtime. This suggests that the intermediate convolutional layers (Conv2, Conv3, Conv4) constitute the main part of the computational resource, and their depth is important for achieving good results. If a relatively lower level of accuracy is acceptable in practical applications, Type-4 architecture would be the best choice.

#### Adjusting the size of each convolutional layers

We then investigated the effects of adjusting the sizes of all convolutional layers except the first one, discussed previously. In Table 2, the filters in each convolutional layer were reduced by 64 each time. Surprisingly, all architectures (Type-8, 9, 10) showed significant decreases in running time with relatively small effects on performance. Especially notable is Type-10 architecture that, with a rather small margin the overall architecture (0.932 vs. 0.917), proceeds about 2.0× faster in training and 1.7× faster in processing rate than the overall architecture. This means redundant filters exist in the intermediate convolutional layers, and a smaller net is sufficient to substitute for the overall architecture, which will enhance the practical utility of the model.

In addition to runtime, another critical component of our models is the ability to implement online learning as accepting unlabeled new samples in the fields. There are multiple components for this process, such as reducing the size of mini-batch (extremity is 1), updating model parameters with samples of low confidence (output of the classification layer)^54^, just retraining the final classification layer, or constructing a sparse auto-encoder to obtain sparse features that allow an effective pre-training on a large dataset consisting of more species as possible (such as additional classes not included in our task) and replacing the model parameters online^49^. Many alternative strategies are available, and evaluation of these alternatives will be the focus of the future work.

### Comparison with Other Methods

In Table 3, we compared our models (Type-1, Type-10) with previous methods on the test set provided by the Department of Plant Protection, Zhejiang University. This dataset contains 50 images for each class, is evenly distributed, thus the mAP (mean average precision) is an indicator of the classification accuracy. We performed this comparison as follows:

#### Comparison with AlexNet

AlexNet^36^ was pretrained on the Imagenet^51^ database and fine-tuned in our experiment. In training and testing with this model, we did not adopt localization but instead resized all the original images to 256 × 256. As shown in Table 3, mAP of AlexNet reaches an accuracy of 0.834. By combining this with saliency map based localization, both our models achieved vastly better performance, 0.923 and 0.951. Obviously, the localization procedure substantially reduced the number of potential false positives in background.

#### Comparison with traditional methods

we selected three traditional methods^20^,^22^,^23^ for comparison with our DCNN pipeline, and have summarized the results and the key techniques for the different methods in Table 3. All models were trained with *Pest ID* images, and evaluated on the localized test images. We found that our method allowed improvement of at least 0.1 over the other models, conforming the effectiveness of DCNNs to extract and organize the discriminative information.

#### The effectiveness of DCNN

**Figure 10:**
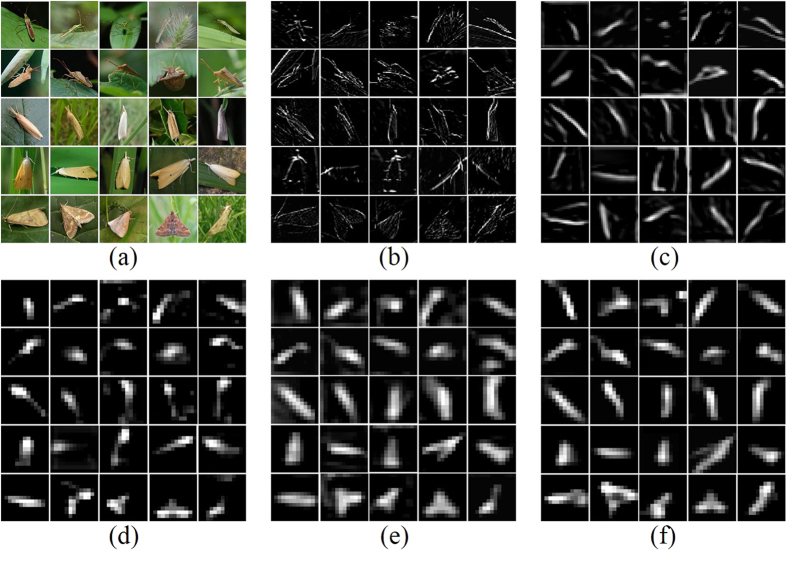
Visualization of feature maps in the overall architecture. (**a**) A subset of original images, which had been performed on a localization processing, were selected from the test set for illustrating pose variations (Row 1&2), inter-species similarity (Row 3&4) and intra-class difference (Row 5). These images were then used in our recognition tasks, and in (**b–f**), we show the top activated feature maps of the corresponding original images after layers Conv1–5. The brightness and contrast were enhanced for each feature map for the best view. Original images are provided by Huan Zhang.

to understand how the steps of our process achieved better performance, we visualized the feature maps with the strongest activation from each layer of the overall architecture to look inside its internal operation and behavior, as shown in Fig. 10. The original images that have been localized are shown prior to the levels of image processing and analysis. Layer 1 responds to edges or corners and layer 2 performs the conjunctions of these edges. Layer 3 allows more complex invariances, capturing distinctive textures. Layer 4 and layer 5 roughly cover the entire objects, but the latter is more robust in distinguishing the objects from the irrelevant backgrounds. The visualization clearly demonstrates the effectiveness of DCNN in handling significant pose variation (rows 1, 2), inter-classes similarity (rows 3, 4) or intra-class variability (row 5).

### Conclusion and future work

We have demonstrated the effectiveness of using a saliency map-based approach for localizing pest insect objects in natural images. We applied this strategy to internet images and constructed a standard database *Pest ID* for training DCNNs. This database has unprecedented scale and thus significantly is enriched for the variation of each species. This allows the construction of powerful DCNNs for pest insect classification. We also proved a large DCNN can retain satisfactory performance with great reduction to its architecture, required for practical application. The pipeline of both localization and classification was not used previously and thus we are the first to report this strategy for a pest insect classification task.

Our approach can be improved further. (1) Including a finer search in the saliency maps may improve the localization accuracy, and is beneficial for expanding *Pest ID* to include significantly more species in the future. (2) Online learning could be implemented to make use of unlabeled new samples in the field for updating the model parameters. (3) The difficulty in interpretation when object overlapping occurs remains a challenge that will need to be addressed to allow the practical application of this design.

## Additional Information

**How to cite this article**: Liu, Z. *et al*. Localization and Classification of Paddy Field Pests using a Saliency Map and Deep Convolutional Neural Network. *Sci. Rep.* **6**, 20410; doi: 10.1038/srep20410 (2016).

## Figures and Tables

**Table 1:** Details of *Pest ID*.

Species	*Cnaphalocrocis medinalis*	*Chilo suppressalis*	*Parnara Guttata*	*Nilaparvata lugens*	*Nephotettix cincticeps*	*Diamondback moth*
Quantity	480	485	481	399	370	554
Species	*Scirpophaga incertulas*	*Oxya chinensis*	*Naranga aenescens*	*Ostrinia rnacalis*	*Sogatella furcifera*	*Cletus punctiger*
Quantity	520	400	381	401	183	482

**Table 2:** Effects of changing the overall architecture on performance and runtime.

Type Index	Architectural Change	Training Time/Speedup	Processing Rate/Speedup	Validation Accuracy
1	Overall architecture	13.9 h/1.0×	3.88 ms/1.0×	0.932
2	Removed layer FC7	12.8 h/1.1×	3.40 ms/1.1×	0.908
3	Removed layers FC6, 7	12.5 h/1.1×	3.05 ms/1.3×	0.897
4	Removed layers FC6, 7, Conv5	10.1 h/1.4×	2.45 ms/1.6×	0.869
5	Removed layers FC6, 7, Conv4, 5	9.5 h/1.5×	1.78 ms/2.2×	0.724
6	Removed layers FC6, 7, Conv3, 4, 5	6.6 h/2.1×	1.66 ms/2.3×	0.633
7	Removed layers FC6, 7, Conv2, 3, 4, 5	3.1 h/4.5×	1.65 ms/2.3×	0.630
8	Adjust Layers Conv2, 3, 4, 5: 192, 320, 320, 192 filters	12.0 h/1.2×	3.48 ms/1.1×	0.929
9	Adjust Layers Conv2, 3, 4, 5: 128, 256, 256, 128 filters	9.2 h/1.5×	2.84 ms/1.4×	0.924
10	Adjust Layers Conv2, 3, 4, 5: 64, 192, 192, 64 filters	7.1 h/2.0×	2.28 ms/1.7×	0.917

The processing rate indicates the time of a feed-forward pass for one image. Speedup denotes the time ratio of the changed architectures versus the overall architecture.

**Table 3:** Comparison of DCNNs with other methods on the same dataset.

Representation	Classifier	Accuracy
Hessian-Affine, SIFT, and shape etc.^20^	SVM	0.610
HOG, SURF^22^	SVM	0.802
Color, Shape and Texture etc.^23^	Fisher	0.817
AlexNet^36^	Softmax	0.834
Type-1 Architecture (overall architecture)	Softmax	0.923
Type-10 Architecture	Softmax	0.951
